# Protective Effect of Human Amniotic Fluid Stem Cells in an Immunodeficient Mouse Model of Acute Tubular Necrosis

**DOI:** 10.1371/journal.pone.0009357

**Published:** 2010-02-24

**Authors:** Laura Perin, Sargis Sedrakyan, Stefano Giuliani, Stefano Da Sacco, Gianni Carraro, Liron Shiri, Kevin V. Lemley, Michael Rosol, Sam Wu, Anthony Atala, David Warburton, Roger E. De Filippo

**Affiliations:** 1 Developmental Biology and Regenerative Medicine Program, Saban Research Institute, Childrens Hospital Los Angeles, Los Angeles, California, United States of America; 2 University of Southern California Institute of Urology, Keck School of Medicine, University of Southern California, Los Angeles, California, United States of America; 3 Wake Forest Institute for Regenerative Medicine, Wake Forest University School of Medicine, Winston-Salem, North Carolina, United States of America; University of Giessen Lung Center, Germany

## Abstract

Acute Tubular Necrosis (ATN) causes severe damage to the kidney epithelial tubular cells and is often associated with severe renal dysfunction. Stem-cell based therapies may provide alternative approaches to treating of ATN. We have previously shown that clonal c-kit^pos^ stem cells, derived from human amniotic fluid (hAFSC) can be induced to a renal fate in an ex-vivo system. Herein, we show for the first time the successful therapeutic application of hAFSC in a mouse model with glycerol-induced rhabdomyolysis and ATN. When injected into the damaged kidney, luciferase-labeled hAFSC can be tracked using bioluminescence. Moreover, we show that hAFSC provide a protective effect, ameliorating ATN in the acute injury phase as reflected by decreased creatinine and BUN blood levels and by a decrease in the number of damaged tubules and apoptosis therein, as well as by promoting proliferation of tubular epithelial cells. We show significant immunomodulatory effects of hAFSC, over the course of ATN. We therefore speculate that AFSC could represent a novel source of stem cells that may function to modulate the kidney immune milieu in renal failure caused by ATN.

## Introduction

Acute Tubular Necrosis (ATN) is characterized by acute tubular cell injury and renal dysfunction [1], [2]. Within the last decade stem cells have emerged as a potential therapeutic tool [4] for acute and chronic kidney disease. Recently, it was shown that mesenchymal stem cells derived from bone marrow (MSC), when injected into mouse models of kidney failure, may contribute to kidney repair either through paracrine effects and/or integration into damaged structures [3]–[6].

In particular, several groups demonstrated that the injection of MSC could help the recovery of Acute Kidney Injury (AKI), when experimentally induced by ischemia-reperfusion injury [7], intramuscular administration of glycerol [5] or by cisplatin [8]. The mechanisms involved in these injuries and the beneficial effects of stem cells still remain controversial and need to be further elucidated.

Atala, his group and we [9] have derived a novel stem cell population from human amniotic fluid that exhibits both embryonic and mesenchymal stem cell characteristics. An important advantage of these cells is that they are easily retrieved through amniocentesis with no injury to the fetus. C-kit^pos^ stem cells derived from amniotic fluid (hAFSC) can be easily propagated *in vitro,* maintaining stem cell-like properties such as pluripotentiality as well as expression of Oct-4, SSEA-4, CD90 and CD105 [9]. In addition, hAFSC have the capacity to differentiate into many different cell types derived from all three germ layers *in vitro* and *in vivo*. They do not form teratomas, and they maintain their telomeric length over many population doublings as well as, normal karyotype [9]. Moreover, we have also demonstrated that when injected into *nu/nu* mice, hAFSC can integrate and differentiate and they express lung epithelial markers following injury. [10].

Our group has also reported the use of amniotic, c-kit^pos^ cells for kidney regeneration [11]. Undifferentiated hAFSC were injected into the kidney of an embryonic mouse in an *ex vivo* culture system and were demonstrated to integrate into the developing organ and participate in all steps of nephrogenesis during development. Herein, we now evaluate the function of hAFSC to rescue damaged kidneys *in vivo* in a mouse model of ATN. In kidneys with ATN caused by glycerol-induced rhabdomyolysis, hAFSC showed the capability to modulate the immune response and restore physiological kidney function parameters to normal. Therefore, we conclude that AFSC represent a new, potentially pluripotential cell source for kidney tissue protection, repair and regeneration.

## Results

### hAFSC Phenotype and Karyotype before Injection

**Figure 1:**
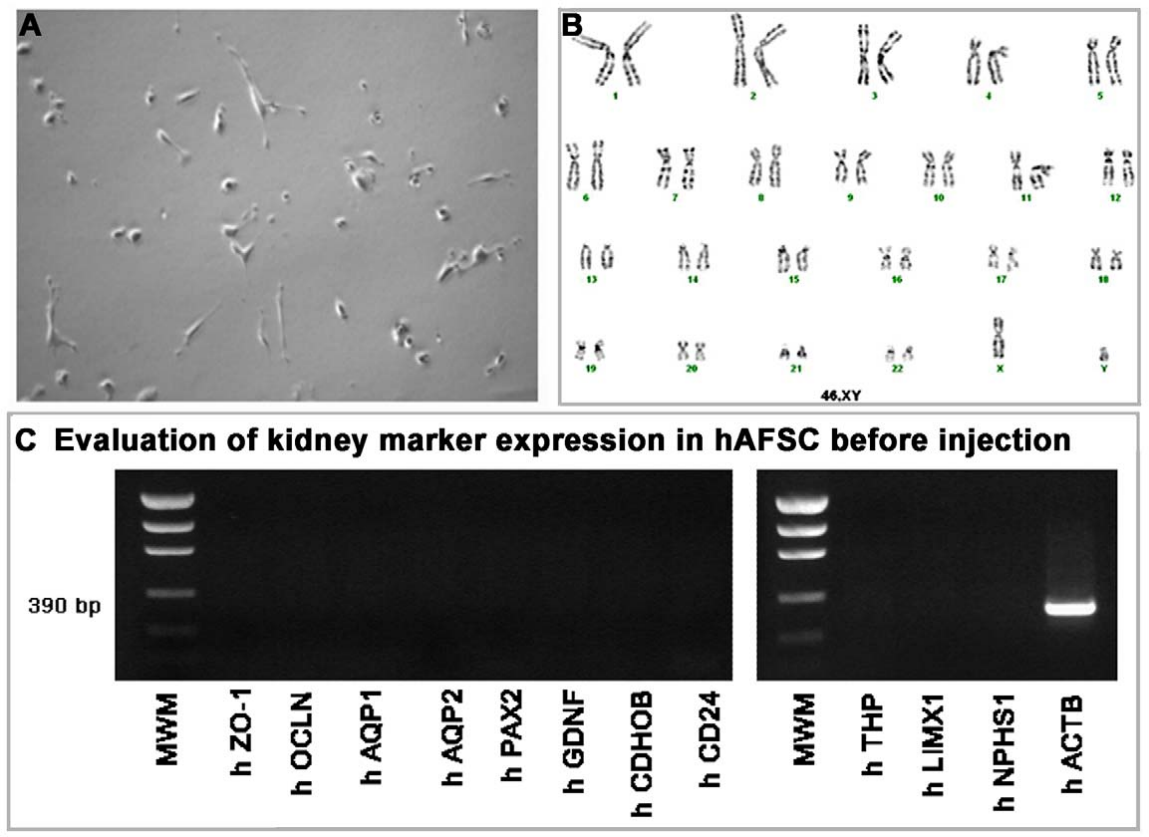
hAFSC morphology, gene expression and karyotype. **A.** Morphology of hAFSC population. After 40 passages in culture under bright field the cells present a fibroblastoid appearance (10×). **B.** Karyotype of hAFSC after 38 passages. **C.** RT-PCR of hAFSC before *in vivo* injection. Neither early nor mature kidney markers are expressed. h*ACTB* is used as a housekeeping gene.

hAFSC, before injection, present a fibroblastoid shape as shown in **Figure 1A**. The cells were then tested to confirm a normal karyotype before *in vivo* applications, to exclude chromosomal abnormalities that could compromise their pluripotential capability (**Figure 1B**). hAFSC were analyzed for the expression of early and late kidney markers before injection. As shown in **Figure 1C**, prior to injection, hAFSC were negative for the most important kidney markers, ranging from transcription factors expressed during early kidney development to late differentiation markers. Thus, we confirmed that hAFSC are not specifically committed to kidney progenitor cells when cultured *in vitro*.

### Evaluation of the Glycerol Induced Muscle Damage and ATN Using Period Acid Schiff Staining (PAS) and TUNEL Staining

**Figure 2:**
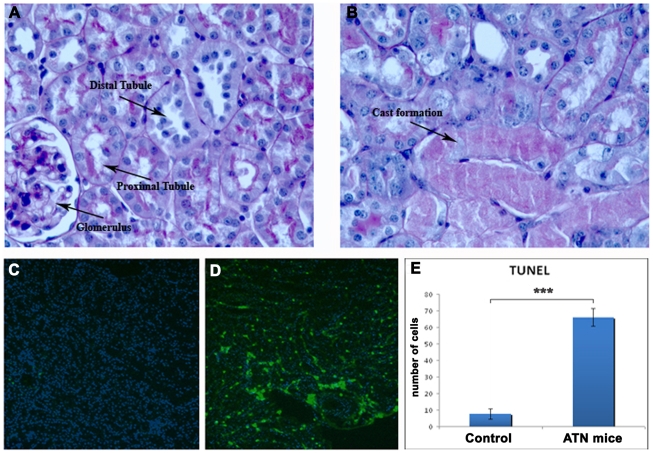
Morphological analysis of glycerol-rhabodomyolysis-induced-ATN model in *nu/nu* mice. **A.** Histological section showing PAS staining of a *nu/nu* mouse kidney. Proximal and distal tubules and glomeruli, as indicated by the arrows, present normal morphology (40x). **B.** Histological section showing PAS staining of a *nu/nu* mouse kidney after 3 days of glycerol-rhabdomyolysis-induced ATN. Destruction of brush borders, intraluminal cast formation as well as general disorganization of the kidney structures is evident (arrow, 40x) **C.** TUNEL staining of a *nu/nu* mouse kidney. The level of apoptotic cells is very low, when compared with TUNEL staining of a *nu/nu* mouse kidney after 3 days of glycerol-induced ATN (**D**), (10x). **E.** Graph showing the effect of glycerol ATN on kidney cell apoptosis (number of positive apoptotic nuclei per 300 nuclei) compared with untreated controls. Values are presented as mean ± SEM (*** p< 0.001).

**Figure 2A** demonstrates normal morphology of a *nu/nu* mouse kidney before any damage; the proximal and distal tubules are intact as well as the glomeruli. **Figure 2B** shows morphology of the kidney 3 days after intramuscular injection of glycerol. Marked disorganization of the normal structure of proximal and distal tubules is evident, with cast formation and brush border disruption, while most of the glomeruli remain intact. This type of damage is typical of ATN injury induced by rhabdomyolysis, where the main structures of the kidneys that undergo damage are tubules and not glomeruli. **Figure 2D** shows an increase in apoptotic cells (TUNEL staining), 72 hours after damage induction as compared with the control that did not undergo glycerol induced muscle damage (**Figure 2C**). The difference in the number of apoptotic cells present in the glycerol treated mice when compared with the untreated control mice was highly statistically significant (P<0.01) (**Figure 2E**)**.**

### *In Vivo* Detection of hAFSC by Bioluminescence

**Figure 3:**
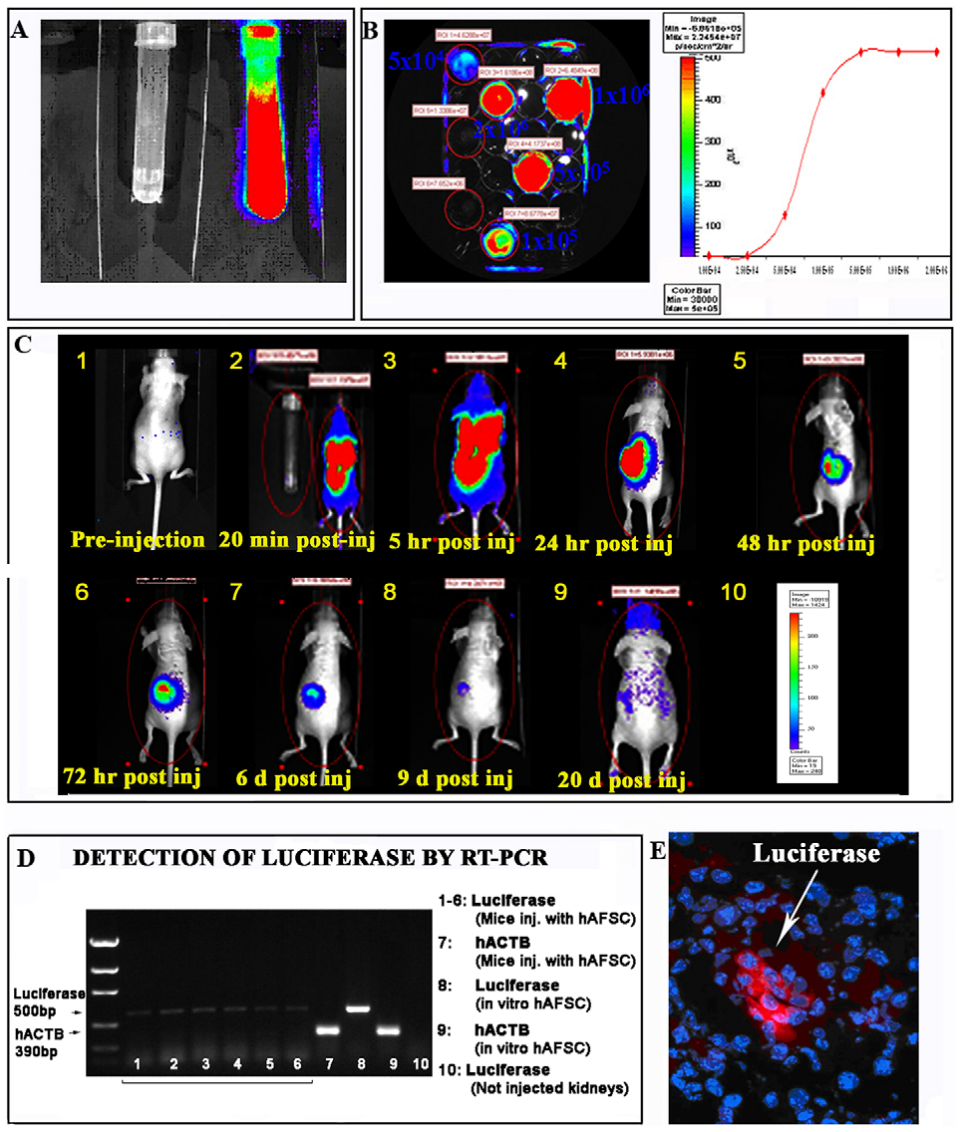
*In vivo* luciferase detection of hAFSC in ATN damaged kidneys. hAFSC transduced with luciferase maintain the expression of luciferase under bioluminescence over many population doublings as demonstrated by the presence of optical signal after 20 passages in culture (tube on right) when compared with hAFSC that were not transfected (tube on left) when stimulated with the substrate for luciferase (**A**). The lowest number of cells that exhibit detectable luciferase signal *in vitro* is 1×10^5^. Luciferase gain range is shown on right (**B**). **C.** *In vivo imaging* showing bioluminescent detection of hAFSC after injection into a damaged *nu/nu* mouse kidney over a period of 21 days. In panel 1 is shown a negative control, injected only with luciferin, the luciferse substrate. The optical signal is strongly present during the first 5 hours post injection (panel 2–5), decreases over time (panel 6–8), but is still present after 21 days (panel 9). In panel 10 is shown the bioluminescence gain range. **D.** RT-PCR demonstrates the presence of the luciferase gene (expressed only by human transfected hAFSC clones) in 5 injected kidneys, compared with the cells before injection (positive control) and in non-injected kidney (negative control). Human *ACTB* is used as housekeeping gene. **E.** Immuno-fluorescence staining of injected kidney with hAFSC after 3 weeks. The red fluorescence (arrow) confirms the presence of hAFSC expressing luciferase. The nuclei are stained with DAPI (20×).

hAFSC, transduced with a lentivirus coding for luciferase, showed stable expression of the transgene over many population doublings. The cells not infected with the lentivirus and exposed to luciferin (the luciferase substate) did not reveal any bioluminescence, while the transfected cells exposed to luciferin continue to express the bioluminescent signal after 20 population doublings (**Figure 3A**).

In **Figure 3B** is shown an *in vitro* experiment to determine the minimal number (1×10^5^) of cells that exhibited a readily detectable optical signal. In **Figure 3C** is shown live imaging of a right kidney injected with 1,2×10^6^ hAFSC after damage induction. The animal injected with luciferin only (as a negative control) did not show any signal (**panel 1**), while the lucierase fluorescent signal is clearly evident, and even spreads into multiple zones of the body, such as the lung over the first few hours (**panels 2–3**). The signal for hAFSC in the area of the kidney can be seen at 24 hours after injection (**panel 4**), was strongest at 48 hours and 72 hours, and persisted for up to 6 days (**panels 5–6**), after which the signal began to diminish over the next several days (**panel 7–8**). However, 21 days after injection, the signal was still evident in the area of the kidney (**panel 9**). Animals were sacrificed at 21 days after injections of hAFSC and DNA extraction and PCR were performed on injected and non-injected kidneys in order to determine the presence of luciferase gene. Both the luciferase gene and a human housekeeping gene *ACTB* (both expressed only in human cells) were present only in the injected kidney tissue, as confirmed by the absence of the both housekeeping *ACTB* and luciferase gene in non-injected kidneys (**Figure 3D**). In addition, the presence of hAFSC in the injected kidneys was also confirmed by positive immunostaining against luciferase, as shown in **Figure 3E** **.**

### Detection of hAFSC in Damaged Kidneys by Immunohistochemistry and Gene Expression

**Figure 4:**
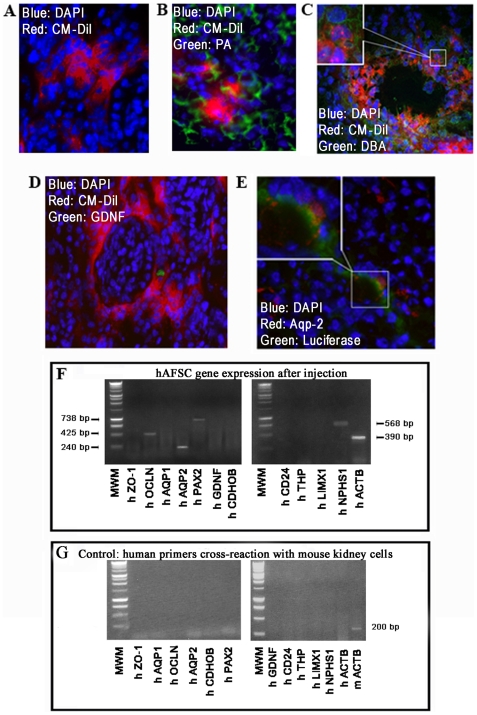
Integration and structural differentiation of hAFSC injected into glycerol induced damaged ATN kidneys. **A.** Frozen section of a kidney injected with hAFSC after 1 week. The cells are evident as red fluorescence of the surface marker CM-DiI. The nuclei are stained with DAPI (30×). It is noticeable that CM-Dil-labelled-hAFSC locate in the proximity of tubular structures after 3 weeks following injection and are shown to express Peanut Agglutinin (**B**) as well as Dolichus Biflorus Agglutinin (arrow) (**C**); hAFSC locate also in close proximity of the glomerular structures and express human Glial Derived Neutrofic Factor (arrow) (**D)**. The nuclei are stained with DAPI (40×). **E.** Double Immuno-fluorescent staining of injected kidney with luciferase transduced hAFSC (and not CM-Dil labeled), showing the same cells expressing both Aquaporin 2 and luciferase (arrow), 3 weeks after injection. The nuclei are, stained with DAPI. (30×). **F.** RT-PCR performed on RNA isolated from kidney, 3 weeks after hAFSC injection. The expressed kidney markers such as *NPHS1*, *AQP2*, *PAX2*, *OCLN*, identified by primers designed with human specific sequences. Human *ACTB* is used as a housekeeping gene. **G** RT-PCR showing the specificity, as indicated by human sequences not cross-reacting with mouse.

The presence of injected hAFSC was evaluated histologically. Frozen sections performed at 1 week after injection confirmed the presence of hAFSC, as detected by red fluorescence of the surface marker CM-DiI (**Figure 4A**). We further observed several instances where the CM-Dil signal from the hAFSC overlapped with the fluorescent-staining of a kidney markers such as Peanut Agglutinin as well as Dolichus Biflorus Agglutinin at 3 weeks after injection; indicating that hAFSC are able to differentiate into cells expressing both adult proximal and distal tubular agglutinins (**Figure 4B,C**). In some rare cases hAFSC were also found in glomerular structures expressing Glial Derived Neurotrophic Factor (GDNF), as identified by a specific antibody reacting against human GDNF, indicating that the stem cells were also able to express early glomerular markers of differentiation (**Figure 4D**). In **Figure 4E**, where the cells were not labeled with the CM-Dil, hAFSC were identified with green fluorescence positive for the luciferase staining, and these injected cells also double stained for Aquaporin 2. After 21 days, RT-PCR was performed using human specific primers on the harvested kidneys, and the expression of several human specific kidney genes (early as well as late markers of differentiation) by the hAFSC in the injected kidneys were identified. Injected hAFSC expressed *NPHS1, AQP2, PAX2*, and *OCLN*, when compared to hAFSC before injection (**Figure 4F**). In **Figure 4G** demonstrates that the human specific primers did not cross react with mouse kidney sequences.

### Kidney Physiology: Serum Creatinine and Blood Urea Nitrogen (BUN) Measurements

A control group of 10 *nu/nu* mice was used to determine basal level of serum creatinine and blood urea nitrogen (BUN) in normal *nu/nu* mice, which averaged 0.6 mg/dl and 27 mg/dl, respectively. After intramuscular injection of glycerol on day 0, creatinine levels increased to as high as 1.10 mg/dl, showing a peak between 48 and 72 hours after injection. Similarly, the level of BUN increased up to 70 mg/dl after glycerol injection and a peak was detected between 48 to 72 hours. The injected dose of glycerol chosen is a sub-lethal dose. Therefore, after the peak, levels of creatinine and BUN resolved back to normal after a period of 3 weeks.

**Figure 5:**
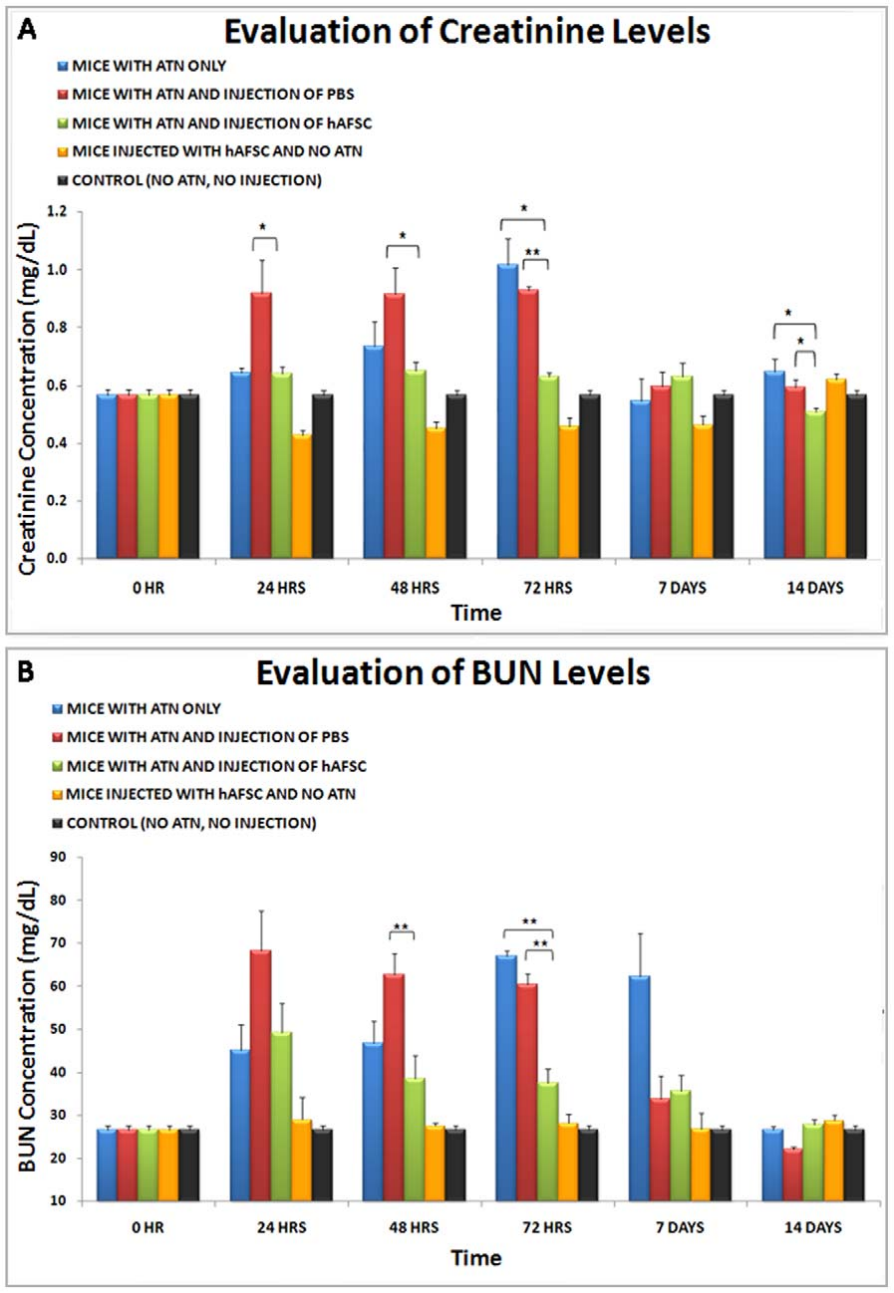
Protective effect of injected hAFSC as determined by measurements of blood creatinine and BUN levels. In these graphs the difference in levels of blood creatinine (**A**) and BUN (**B**) between the experimental groups are shown (Blue: animals that underwent only ATN; Red: animals that underwent ATN and intrarenal injection of PBS; Green: animals that underwent ATN and intrarenal injection of hAFSC; Orange: animals that underwent intrarenal injection of hAFSC but not ATN; Black: control animals, no ATN and no injections), shown at different time points (24 hours, 48 hours, 72 hours, 1 week and 2 weeks). Values are presented as mean ± SEM (* p<0,05; ** p<0,01).

Remarkably, animals subjected to damage induced with glycerol and receiving an intrarenal hAFSC injection demonstrated no increase in the levels of creatinine or BUN during the expected acute phase of injury, showing a statistically significant difference when compared with animals treated with PBS after ATN or with animals that underwent only ATN induction. Absence of increases in both creatinine and BUN levels was demonstrated also in animals that received only intra-renal injection of hAFSC without glycerol damage, demonstrating that hAFSC alone did not alter these kidney physiological parameters over time (**Figure 5A,B**).

### Morphological Studies

**Figure 6:**
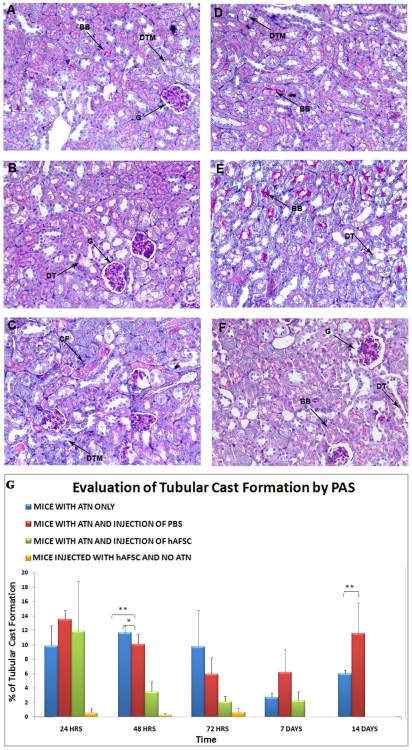
Protective effect of injected hAFSC, determined by maintenance of the morphological structure in glycerol induced ATN damaged kidneys. In these figures are shown the most representative PAS-Staining of kidney sections of mice treated only with injection of glycerol over the course time of 24 hours (**A**), 48 hours (**B**) and 72 hours, (**C**) when compared with mice treated with injection of glycerol and hAFSC at 24 hours (**D**), 48 hours (**E**) and 72 hours (**F**) after injection. In the mice treated only with glycerol the level of disruption of brush border (BB), the desegregation of tubular membrane (DTM) and cast formation (CF) increased over time, while the injection of hAFSC preserved the morphology of the tubular structures after they have been damaged following IM glycerol injection. The graph (**G**) represents the percentage of damaged tubules in the different experimental groups. Blue: animals that underwent only ATN; Red: animals that underwent ATN and intrarenal injection of PBS; Green: animals that underwent ATN and intrarenal injection of hAFSC; Orange: animals that underwent intrarenal injection of hAFSC but not ATN) at different time points (24 hours, 48 hours, 72 hours, 1 weeks and 2 weeks) per total number of tubules in the sections. Values are presented as mean ± SEM (* p<0,05; ** p<0,01).

In **Figure 6** is shown an example of PAS histological staining of a typical animal treated with hAFSC after ATN induction, compared with another typical animal that underwent only ATN (Figure 6A-C), both sampled during the expected peak of IM glycerol induced ATN damage at 48–72 hours. In particular, in **Figure 6A–C** an increase in the number of damaged tubules is seen from 24 hours to 72 hours in the glycerol-treated control animals. By 72 hours the damage appears to be more severe due to cast formation (arrow, **Figure 6C**) within damaged tubules. In **Figure 6D-F** it is shown that in glycerol-injected animals treated with hAFSC, a number of damaged tubules is present at 48 hours, but by 72 hours the number of damaged tubules and the cast formation within them has decreased significantly. The evaluation of tubular injury, based on counting cast formation in different experimental groups at different time points (24 hours, 48 hours, 72 hours, 1 week and 2 weeks) showed that animals that were treated with hAFSC exhibited less damage during the peak phase, compared to the animals that were not treated with hAFSC, or to those treated with saline vehicle solution (PBS). In addition, animals that underwent only intrarenal injection of hAFSC did not show any cast formation (**Figure 6G**).

Animals that did not undergo any treatment at all (normal control animals) did not show any cast formation at basal level (data not shown), and were therefore not included in the statistical analysis comparing ATN groups of animals.

### Proliferation (PCNA Staining) and Apoptosis (TUNEL Staining)

The proliferative activity and the apoptosis of tubular cells were compared between the experimental groups at the different time points (24 hours, 48 hours, 72 hours, 1 week and 2 weeks).

**Figure 7:**
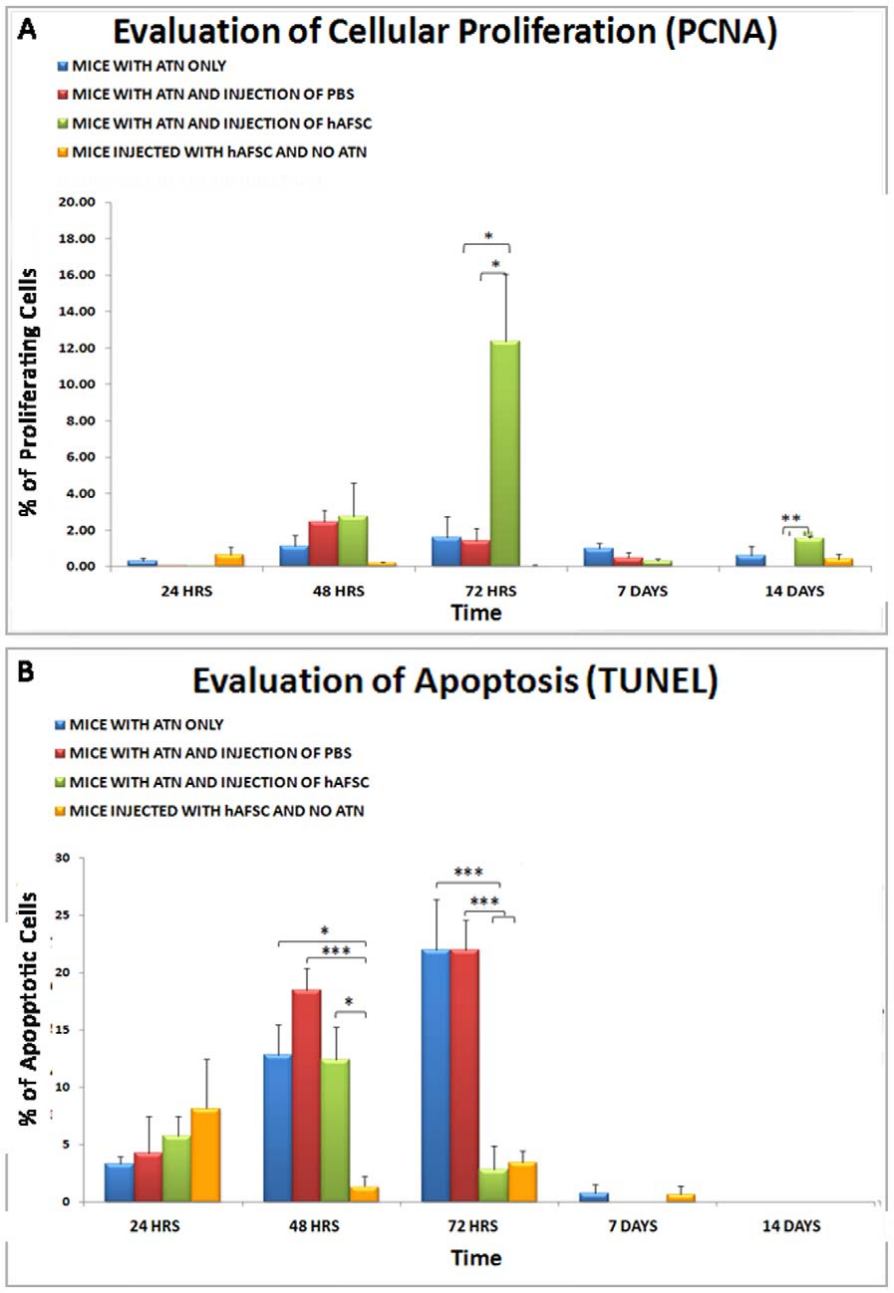
Protective effect of injected hAFSC as determined by increase of proliferation and decrease of apoptotic tubular cells in glycerol induced ATN kidneys. In these graphs is shown the proliferation activity in tubular cells (**A**) and the apoptosis (**B**) between the experimental groups (Blue: animals that underwent only ATN; Red: animals that underwent ATN and intrarenal injection of PBS; Green: animals that underwent ATN and intrarenal injection of hAFSC; Orange: animals that underwent intrarenal injection of hAFSC but not ATN;) at different time points (24 hours, 48 hours, 72 hours, 1 week and 2 weeks). Values are presented as mean ± SEM (* p<0,05; ** p<0,01; *** p<0,001).

The analysis of the data showed no statistical difference in proliferation activity between the animals that were injected in the kidney with saline vehicle solution (PBS) after glycerol injection versus animals that underwent only glycerol injection over time. In contrast, animals subjected to damage induced with glycerol and that received intrarenal hAFSC injection demonstrated a significant increase in cell proliferation between 48 hours and 72 hours (**Figure 7A**).

Apoptosis increased in animals that were injected with saline vehicle solution (PBS) after glycerol injection as well as in animals that underwent only glycerol injection, as measured during the acute phase of injury. In contrast, in animals that received intrarenal injection of hAFSC a decrease of apoptosis was noted, as shown in **Figure 7B**. The absence of increase in proliferation or apoptosis was demonstrated in animals that received only intrarenal injection of hAFSC without glycerol damage, demonstrating that hAFSC alone do not adversely influence normal tubular cells (**Figure 7A,B**). Animals that did not undergo any treatment (control animals) did not show any remarkable apoptosis or proliferation (data not shown), and were therefore not included in the statistical comparison between ATN groups.

### Immuno-Cytokine Profile

**Figure 8:**
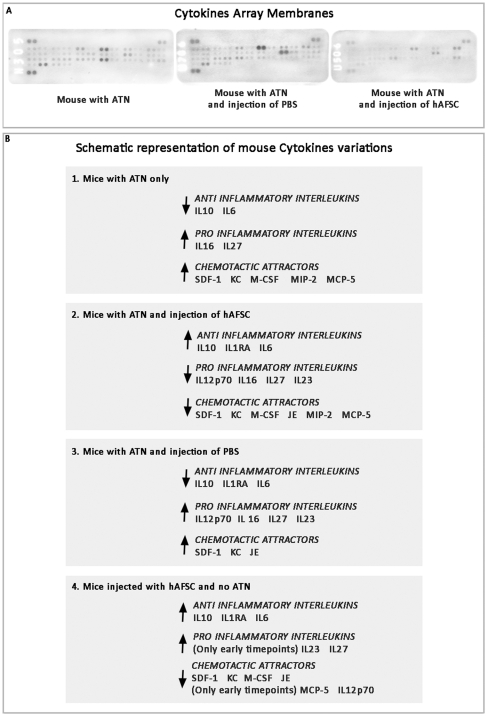
Immunomodulatory effects of hAFSC once injected into glycerol induced ATN-damaged kidneys. Mouse Cytokines were analyzed among all the experimental groups to evaluate changes in the inflammatory milieu. **A.** Developed multiplex cytokine assay membranes for mouse with ATN, mouse with ATN and Injection of PBS and Mouse with ATN and injection of hAFSC, showing the markedly different expression patterns of cytokines at 14 days. **B.** Schematic outline representation of mouse cytokine expression over the 14 days among different groups: 1. Mice with ATN only. As a general trend of cytokine expression, in these animals the pro-Inflammatory cytokines increased or remained highly expressed and anti-inflammatory cytokines showed decreased or low expression; 2. Mice with ATN and injection of hAFSC. As a general trend of cytokine expression, it is evident that the anti inflammatory cytokines increased over the 14 day study period, while pro-Inflammatory cytokines decreased; 3. Mice with ATN and injection of PBS. As a general trend of cytokine expression, in these animals the pro-Inflammatory cytokines increased or remained highly expressed and anti-inflammatory cytokines showed decreased or low expression; 4. Mice without ATN and injection of hAFSC. As a general trend of cytokine expression, in these mice the anti-inflammatory cytokines are mildly increased over the 14 days, while pro-inflammatory cytokines are not expressed or show decreased expression over the 14 days.

Since the salutary effect of hAFSC injection occurred during the acute phase of ATN, we postulated that this protective effect might involve acute changes in the kidney's cytokine expression. Cytokines expression arrays were performed using Cytokine Array Membranes (**Figure 8A**). For relative ease of interpretation, the different cytokines are displayed as five broad functional clusters, based on their principal immunological functions: **1.** Interleukins; **2.** Activators of B Lymphocytes; **3.** Activators of Natural Killers; **4.** Chemotactic attractors of Granulocytes and Macrophages; **5**. Multiple biological effectors. We did not include a specific category for activators of T Lymphocytes, since in this mouse model activated T Lymphocytes are not expressed. We analyzed the expression of cytokines in all the experimental groups at different time points up to two weeks. The mouse specific cytokine assay does not cross react with the human cytokine assay. This was confirmed by incubating digested kidney extracts with membranes specific for human cytokines and conversely incubating hAFSC with membranes specific for mouse cytokines (data not shown).

Mice treated with intrarenal hAFSC after induction of ATN showed statistically significant comparisons, mostly at 7 and 14 days, in mouse cytokine levels, when compared with mice treated with either intrarenal vehicle saline solution (PBS) or just with ATN induction alone, or when compared with *nu/nu* mouse kidney before any treatment, as shown in **Table S1** and **Table S2** (Supplementary Data). There was no significant difference, with very few exceptions, between the ATN mice treated with vehicle solution (PBS) versus just ATN induction, as shown in **Table S2**. In addition, the levels of cytokines in the hAFSC treated ATN mice, compared with basal *nu/nu* mouse kidney levels showed that there was an increase of anti-inflammatory cytokines such as IL-10 and IL-1Ra (**Table S1**). Moreover, there was no significant increase in pro inflammatory cytokines such as IL 27, IL 12p70, IL 2, IL 23 (**Table S1**)**.** Indeed the hAFSC injected ATN mice showed a decrease in JE, M-CSF, MIP-2, MPC-5 and KC expression, when versus the ATN mice treated with vehicle solution (PBS) or just ATN induction (**Figure 8B** and **Table S2**)**.**

Mice that were injected with hAFSC, only without inducing ATN, underwent a limited tissue reaction in terms of production of different cytokines, but for most of them this expression was very early after the injection (within the first few days) and the levels returned to normal by 14 days as shown in **Table S1** and **Figure 8B**. When the human cells are injected (both in mice with or without ATN) there was a noticeable increase in human cytokine levels, when compared with the cytokines expressed by hAFSC before injection (*in vitro* culture) as reported in **Table S3** especially during the first days after injection, while most of them returned to normal at 7 days and were not detectable at 14 days.

## Discussion

In this paper we demonstrate a protective role of hAFSC when injected directly into *nu/nu* mouse kidneys with glycerol-induced ATN. This model of ATN involves a complex sequence of events wherein myoglobin, released from damaged muscle, damages the epithelial cells of the proximal tubules, producing cast formation, vasoconstriction and decreased glomerular filtration. Peak of the damage represented by high levels of apoptotic cells, increased levels of creatinine and BUN and by histological analysis was confirmed to be between 48–72 after induction of ATN.

The ability for hAFSC to home to injured organs was confirmed using luciferase positive hAFSC detected their bioluminescence after injection. As shown in our previous paper [10] the amount of hAFSC detected by bioluminescence in *nu/nu* mice, wherein the acute injury was performed in the lung instead of the kidney, decreased over time, showing a strong signal in the lung at the beginning that fades over the next few days. Nevertheless herein following intrarenal injection after ATN kidney damage, the bioluminescent signal is still present in the kidney after 21 days and hAFSC were found specifically located among the tubules. In order to determine if the injected cells can differentiate into tubular epithelial cells we performed both immunohistochemistry and RT-PCR using human specific antibodies and human primers. Genes such as *PAX2*, *NPHS1* and lectins like Dolicholus Biflorus and Peanut Agglutinin are expressed by injected cells, indicating that at least some of the integrated cells are able to commit toward renal differentiation. Furthermore, in some rare instances, injected hAFSC cells could express human Glial Derived Neurotrophic Factor (*GDNF*), which is expressed during very early kidney development; *GDNF* is not usually detectable in the adult kidney, indicating that injected hAFSC can express also embryonic renal markers, retaining the ability of going through the nephrogenesis. We have previously shown that hAFSC have the potential to integrate into embryonic kidneys and can participate in key steps of nephrogenesis, indicating that hAFSC can be induced toward to a renal fate when placed in an appropriate environment [11]. However, both in this study as well as in this previous study, the efficiency of integration into kidney lineages was not strikingly high and we did not detect significant clonal expansion of integrated hAFSC. We, therefore, postulated that perhaps another benefit could be afforded the injured kidney than simply a structural one by the presence of hAFSC during the acute phase of injury.

We determined that hAFSC have the ability to modulate kidney function during ATN, as reflected in serum creatinine and BUN levels. However, the timing of hAFSC is critical: hAFSC injected during the acute phase of ATN (between 48–72 hours after the IM injection of glycerol), had no effect on creatinine and BUN levels (data not shown), and implies that if injury is already established the damage is not attenuated. In contrast, hAFSC injected into the kidney on the same day of glycerol injection resulted in no observed peaks in creatinine or BUN. This beneficial effect with hAFSC was also correlated with significant increases in proliferative activity of tubular epithelial cells, decreased cast formation, and decreased apoptosis of tubular epithelial cells.

We therefore speculate that hAFSC can, when injected early enough (in this study contemporaneously with the time of injury) attenuate acute renal damage, underscoring the potential protective effects of hAFSC. Moreover, even 14 days after injury hAFSC injection into the kidney still correlated with increased tubular cell proliferation and less tubular damage. Thus, we hypothesize that hAFSC might accelerate the proliferation of epithelial tubular cells that were only partially damaged, while in addition preventing apoptosis. This mechanism of protection therefore appears to lead to better maintenance of tubular structure, as seen in PAS staining, thus avoiding the increase in BUN and creatinine typically seen in IM-glycerol-induced ATN.

During acute renal injury the immune response plays a key role. Damaged kidney endothelial cells attract leukocytes, vasomediators are released with injury, and epithelial cells of the tubule produce pro-inflammatory and chemotactic cytokines [12]. Bonventre *et al.* [13] and Lin *et al.* [14] have shown recently that the mechanism by which bone marrow stem cells contribute to renal repair was by attenuating the immune response, rather than through integration or differentiation of the stem cells into the cells of the damaged organ. They also speculated that the protection in these animals was not through integration and differentiation of the injected MSC, because of the very short period of time with which a protective response was observed [15].

The animal model used in these experiments (Athymic Nude-*Foxn1^nu^*) was chosen in order to be able inject human derived stem cells and evaluate their effect over time, avoiding outright rejection. *Nu/nu* mice are immunodeficient, lacking activated T Lymphocytes but not their precursors. They have normal B Lymphocytes and they have evidence of increase numbers of NK cells. Thus, even though they are immunodeficient, they do possess some ability to mount and modulate a partial immune response when stimulated [16]. In addition to the animals that underwent only ATN and mice with no treatment, we introduced mice that were injected with a vehicle saline solution (PBS) after glycerol damage and compared their respective cytokine levels. Therefore, to further investigate the potential mechanisms by which hAFSC enhance renal protection, we examined intrarenal cytokines, to determine whether there is a general change in inflammatory cytokine pattern in mice that were treated with hAFSC compared to our controls. We decided to evaluate the immune response in a time period extending up to two weeks, instead of only the first 3 days, because even if this is an acute injury model, the response of *nu/nu* mice with glycerol induced ATN to human stem cells after kidney injection has never been demonstrated before, and the response could diverge significantly from wild type mice injected with mouse-specific stem cells.

The kidneys of the ATN mice treated with hAFSC presented a profile of mouse cytokines that indicated a lower expression of pro-inflammatory molecules, as compared to mice treated with either ATN plus PBS or that underwent only ATN, versus the basal level in mice before any treatment. One possible explanation for this result may be the increased presence of IL-10 and IL1Ra, both of which are anti-inflammatory cytokines, in the mice treated with hAFSC cells. Moreover, the presence of both of these two anti-inflammatory interleukins appears to have stimulated the production of IL-6, which also possess anti-inflammatory properties in the presence of increased levels of IL-10 and IL1Ra [17]. In mice with ATN and treated with PBS or only with ATN, there is no change in expression of IL-10 and IL-6. Therefore we conclude that inflammation can persist over a longer period of time in these non-hAFSC treated mice. Furthermore, mice treated with hAFSC did not show increased levels of important pro-inflammatory cytokines, such as IL27, a Natural Killer cell attractor [18] or IL12p70, which is known to be inhibited by IL-10 [19], [20]. Furthermore, the injection of the cells prevented an increase in SDF-1 (Stromal cell-Derived Factor-1) a potent B chemoattractor, which is produced by several cell types [21], thus indicating that many different immune functions can be slowed down or controlled by injection of hAFSC. Specific chemoattractants such as KC (Keratinocyte Chemoattractant), JE/MCP-1 (Monocyte Chemoattractant Protein 1), MCP-5 (Monocyte Chemoattractant Protein 5) and MIP-2 (Macrophage Inflammatory Protein 2) are also diminished in mice treated with hAFSC.

It is beyond the scope of the current study to define the specific role of each individual cytokine involved in the immune response in kidney injury and repair. Nevertheless, the total effect of the cytokines and chemokines expressed in the ATN kidneys of mice treated with hAFSC evidently lead to a combined action helping to ameliorate the acute phase of injury.

It is also important to note that in ATN mice that were injected with hAFSC, all cytokines (pro and anti-inflammatory) had returned to normal by 14 days, particularly when compared with ATN mice injected with PBS or with just ATN, i.e., in the latter mice, inflammation persists longer. This is also in accordance with the presence of tubular damage in the latter groups at 14 days. This suggests that an important function of hAFSC may be to actually prevent the acute injury. We further speculate that, in this particular *nu/nu* model of ATN, the beneficial down-modulation of the most important immune cytokines may actually be relatively delayed, because *nu/nu* mice, lacking activated T lymphocytes, can induce alternative pathways of immune responses. One great practical advantage of studying the impact of hAFSC in the *nu/nu* model of ATN is that we could measure and compare the cytokines expressed by the human cells versus those the recipient mouse kidney. Interestingly, human cytokine levels (compared with the basal level secreted in the hAFSC supernatant *in vitro* before injection) are significantly increased early in the course of ATN (as shown Table S3), while the mouse cytokines are only significantly increased after 1 or 2 weeks. Thus the human cells are able to produce cytokines that can modulate the *nu/nu* mouse response to ATN.

With a few exceptions, most human cytokines are also active on mouse cells [22]–[27], so both the combined effects of human and mouse cytokines may have affected the kidney inflammation and tissue homeostasis milieu. We think that this may be an important concept, and therefore consider that maybe cytokines derived from both the injected hAFSC and the endogenous mouse cytokines are responsible for the observed protective effects. In addition, it is relevant to underline the decrease in number of injected hAFSC found in the kidney over time, as shown both by the bioluminescence images and by the immunohistochemistry data, as well as by the absence of human cytokine expression at 14 days. What is essential to underline in this work and in this mouse model of ATN is that, in *nu/nu* mice treated with hAFSC, there is over all improvement in the maintenance of tissue homeostasis, which prevented progression of the acute phase in glycerol-induced ATN, most likely through stimulating proliferation of tubular epithelial cells and by cytokine-mediated paracrine mechanisms. Furthermore, after the acute phase, the decrease of creatinine levels, as well as less cast formation and the increase of tubular cell proliferation is still statistically significant at two weeks, at which time damage still persists in the ATN mice treated only with PBS or not treated at all. This is also in accordance with the data on cytokine levels that are still significantly elevated at 1 and 2 weeks in ATN mice treated with PBS only or not treated at all, as compared to ATN mice treated with hAFSC in which cytokine levels had resolved by 1–2 weeks.

To rule out whether hAFSC alone can cause damage to the kidney and/or produce cytokines once injected *in vivo,* we injected them into *nu/nu* mice without ATN induction. In these mice, injected only with hAFSC there was no alteration of creatinine or BUN, no increase in apoptosis or proliferation or tubular damage; thus indicating that hAFSC per se do not alter the normal physiology and morphology of the kidney. However injection of hAFSC alone did stimulate the production of some cytokines by *nu/nu* mouse kidney, but their levels were relatively low and only detectable predominantly during the first few time points, and rapidly resolved back to normal before 14 days, as compared with ATN mice injected with PBS. In addition, in these mice, there was an increase of levels in IL-10 and IL-1Ra and IL-6 following hAFSC injection (similar to that noted in the mice injected with hAFSC following ATN), there was no increase in SDF-1 and also they expressed lower levels of NK attractors as well as macrophage attractors. Thus, hAFSC do stimulate a modest tissue reaction by *nu/nu* mice, but this did not cause any measurable perturbation of kidney function. In addition, the human cytokines produced by the injected cells in mice that did not undergo ATN disappear quickly and with no apparent adverse consequences.

In conclusion, we have demonstrated that early direct injection of hAFSC into the kidney strongly ameliorates ATN injury, as reflected by more rapid resolution of tubular structural damage, by tubular cell proliferation and by normalized creatinine and BUN levels. In addition, our data show evidence of potent immunomodulatory effects of hAFSC that appear to control the local immune response in favor of a tissue cytokine and cellular milieu that promotes prevention or resolution of tissue damage. Taken together these findings suggest that hAFSC may have therapeutic potential in ATN and, by extrapolation, perhaps in other kidney diseases.

## Materials and Methods

### Isolation and Labelling of hAFSC

Samples of human amniotic fluid from male fetuses (12–18 week of gestation) were provided to our laboratory by Genzyme Genetics Corporation (Mongovria, CA, USA) after karyotyping analysis. No written or verbal consent was required since samples were not identified and information obtained about the samples was limited to karyotype and fetal health status. The stem cell population was separated from the general human amniotic cellular milieu using standard Magnetic Sorting (MACS) techniques [Miltenyi Biotech] against the cell surface marker, c-kit, as described by Atala *et al*. [9]. Pluripotential characteristics of the clonal and subclonal groups were tested according to protocols also outlined by Atala *et al*. [9]. The isolation and characterization of pluripotency of human and mouse AFSC is a very well established protocol in our laboratory, and clones used in these experiments are the same as used for our previous publications [10], [11]. Expression of pluripotent markers such us Oct-4, stage-specific-embryonic antigen 4 (SSEA-4), CD90 and CD105 were confirmed using FACS (data not shown) in order to confirm their phenotype described in the original paper [9]. Clones, derived from a single sample of human amniotic fluid, were then cultured in petri dishes in medium containing α-MEM Medium [Gibco/BRL] supplemented with 20% Chang Medium B [Irvine Scientific] and 2% Chang Medium C [Irvine Scientific], 20% Fetal Bovine Serum [Gibco/BRL], 1% L-Glutamine [Gibco/BRL], and 1% antibiotics (pen-strep) [Gibco/BRL]. hAFSC, used for *in vivo* injection, were rekaryotyped at passage 38 using standard protocols by the Core Laboratory of Clinical Cytogenetics directed by Dr Wu at Childrens Hospital Los Angeles.

Before injection, a clonal hAFSC population at passage 40 was trypsinized in 0.05M trypsin/EDTA [Gibco/BRL] solution and centrifuged at 1500 rpm for 5 min, and then labeled with a cell surface marker CM-DiI [Molecular Probe] following the manufacturer's instructions, in order to track the cells after injection. Briefly, the cells were incubated with a working solution of 1 mg/ml of CM-Dil for 5 minutes at 37°C followed by an incubation of 15 minutes at 4°C and then three washes with phosphate-buffered saline (PBS) [Gibco/BRL].

### ATN Induction and Injection of hAFSC

Rhabdomyolysis-related ATN was induced in female *nu/nu* mice [Jackson Laboratories] by intramuscular injection with 50% hypertonic glycerol solution in water (10 ml/kg body/wt) [Sigma-Aldrich] following deprivation of water for 22 hours. Controlled intramuscular injection of glycerol was performed under anesthesia by surgically exposing the caudal thigh muscle and slowly injecting the glycerol solution prior to delivery of cells. In *nu/nu* mice the amount of glycerol required for induction of ATN was 50% higher than the dose needed in wild type mice (data not shown). This suggests that the T-deficient mice are relatively protected as compared to wild type mice against glycerol-rhabdomyolysis-induced ATN [28].

Animal experiments were performed in adherence to the National Institutes of Health Guide for the Care and Use of Laboratory Animals, with institutional Animal Care and Use Committee approval. The mice were carefully anesthetized using isofluorane inhalation. Once satisfactory anesthesia was achieved, the mice were prepared for surgery using chlorhexidine. A 1 cm dorsal incision was made, both kidneys were carefully delivered via the incision, and the labeled hAFSC (1×10^6^ resuspended in 50 µl of saline vehicle solution, PBS) at passage 40 were carefully injected into the renal cortex of both kidneys with a 30–33 gauge needle, using a microinjector Eppendorf TransferMan NK2 Injector [Eppendorf], 2 hours after intramuscular glycerol injection. The kidneys were then replaced into the retroperitoneum, the incision closed with polypropylene suture and the mice were allowed to recover from anesthesia. The animals were maintained on a heating pad throughout the period of anesthesia. 0.1 mg/kg of buprenorphine was administered subcutaneously and 1 mg/kg bupivicaine (a local anesthetic) along the incision margins just prior to wound closure to provide post-operative pain relief. The animals were sterilely draped to prevent contact of the kidneys with the skin of the animal to reduce risk of peritonitis. As control, mice were injected with saline vehicle solution (PBS) 2 hours after glycerol damage. In addition, another animal group was injected only with hAFSC without previous glycerol damage using the same technique described above for the cell suspension and surgery.

### Tissue Processing

At different times points (from 24 hours to 3 weeks), the injected and the control mice were sacrificed. The kidneys were extracted, washed in PBS, and processed in one of the following ways depending on the analysis performed.

#### 1. RNA/DNA extraction

The kidneys were minced in small pieces and the RNA extracted using Qiagen RNeasy kit [Qiagen] according to the manufacturer's instructions. Total RNA was isolated using the RNeasy Mini Kit [Invitrogen] as described on the data sheet. Briefly, with the use of silica-gel columns RNA is separated from DNA through centrifugation after lysis and homogenization of the samples. Ethanol addition allows RNA to bind the silica-gel before the centrifugation step. The RNA solution obtained was then processed with DNAse treatment [DNAse I, Invitrogen] to avoid any possible genomic contamination. 1 µg of total RNA was reverse transcribed using SuperScript II reverse transcriptase [Invitrogen]. Amplification of the resulting cDNA was carried out using only specific human primers not coding for mouse sequences. A PCR thermal cycler [Eppendorf] was employed after an initial denaturation step at 95°C for 10 minutes. We used a denaturation step at 95°C for 30 seconds, an annealing step at the temperature specific for each primer (ranging from 54°C to 60°C) for 45 seconds, and an extension step at 72°C for 45 seconds for a total of 35 cycles. Detection of the PCR amplification products was performed by size fractionation on 1% agarose gel electrophoresis. As a housekeeping gene, amplification of fragments of the human β-actin RNA was performed. Specific human primer sequences, predicted sizes of amplicons and specific annealing temperatures are shown in Table 1.

**Table 1:** List of the human primers, the size of the products and the annealing temperature used in the experiments.

Gene	Primer Sequenze (5′→3′)	Size (bp)	Annealing Temperature
Nephrin, *NPHS1*	aca cgg agc aca cat acc ac gga ttg gag agg agc aga ag	570	59
Zona Occludens-1, *ZO1*	agg aga ggt gtt ccg tgt tg gct ggt ttt gct gtt gtt ga	760	59
Glial Derived Neurotrophic Factor *GDNF*	tat ggg atg tcg tgg ctg t aca cct ttt agc gga atg ctt	630	58
Aquaporin-1, *AQP1*	cac ctc ctc cct gac tgg ggt tgc tga agt tgt gtg tga	290	58
Aquaporin-2, *AQP2*	gat cac gcc agc aga cat c ggg cag gat tca tag agc ag	240	59
Tam-Horsfall-Protein, *THP*	tag acg agg act gca aat cg gtc ccg gtt gtc tct gtc at	220	59
OB-Cadherin, *CDHOB*	Cactgtctttgcagcagaaatc tacaatgaccaaggagaatgacg	430	55
*CD24*	acc cag cat cct gct aga c ctt aag agt aga gat gca gaa	290	59
LIM1, *LIMX1*	aag agc gag gat gaa gat gc tca gga ggc gaa gtagga ac	620	59
Occludin, *OCLN*	gcc ctc gca acc caa att tta tca ttc act ttg cca ttg ga	430	58
PAX-2, *PAX2*	aac gac aga acc cga cta tgt t agg atg gag gga cca act gc	740	59
Beta-actin, *ACTB*	aga aaa tct ggc acc aca cc ctc ctt aat gtc acg cac ga	390	55
Luciferase	agg agc ctt cag gat tac aag att caa agt gta ctt aat ca gaga ctt cag gcg ggt caa c	500	58

In order to perform PCR on the genomic DNA to evaluate the presence of the luciferase gene, DNA extraction was performed following standard protocols of the Qiagen DNeasy kit [Qiagen,]. Briefly, the kidney was cut in small pieces and incubated at 55°C with proteinase K until completely lysed. To the mixture was then added buffer AL and ethanol 70% furnished by the kit to obtain ideal DNA binding conditions to the DNeasy Mini Spin column. After centrifugation and washing steps DNA was collected and PCR was performed as previously described.

#### 2. Histology

Kidneys were fixed in 4% paraformaldehyde[Sigma-Aldrich] in PBS for 8 hours at 4°C, dehydrated through a gradual series of alcohol, embedded in paraffin, and sectioned at 4–5 µm. The sections were deparaffinized for 15 minutes in histochoice [Sigma-Aldrich], 5 minutes in 100% alcohol, 5 minutes in 95% alcohol, 5 minutes in 70% alcohol; 5 minutes in 50% alcohol, 5 minutes in 30% alcohol and then water. The sections were then stained with Periodic Acid Schiff (PAS) [Sigma-Aldrich] to evaluate kidney morphology. The slides were immersed in Periodic Acid Solution [Sigma-Aldrich] for 5 minutes at room temperature; rinsed in several changes of distilled water, then stained with Schiffs reagent [Sigma-Aldrich] for 15 minutes at room temperature, washed in running tap water for 5 minutes and finally counterstained with hematoxylin solution Gill [Sigma-Aldrich] for 90 seconds. In the end the slides were rinsed in running tap water, dehydrated, cleared and mounted in mounting medium PROTOCOL XYLENE BASED [Fisher Scientific].

In addition, some kidneys were frozen in liquid nitrogen using Tissue-Tek O.C.T. compound [Finetek] and stored at −20°C. When required, kidneys were cryosectioned at 5 µm and then used for immuno-histochemistry.

### Transduction of the AFSC with Luciferase Gene: Bioluminescent Detection

Clones of hAFSC were transduced with a lentiviral vector (SMPU-R-MNCU3-LUC based on HIV-1 that transduces the firefly luciferase gene) made by the Vector Core Facility at Childrens Hospital Los Angeles following standard protocols as reported in the paper by Crooks *et al* [29]. The Vector Core performed the transduction, the titration of the virus and the multiplicity of infection. Briefly, two cycles of transduction were performed by removing old medium and adding new virus supernatant and medium. 24 hours after the initial transduction, cells were thoroughly washed 3 times with PBS before transplantation or *in vitro* analysis. The percentage of the transfected cells was 40%; positive clones were selected and used for the experiments. Before *in vivo* injections, a simple *in vitro* test was employed to determine the minimum amount of hAFSC detectable by bioluminescence. Different concentrations of the cells ranging from 5×10^4^ to 2×10^6^ were evaluated. In addition, the expression of the luciferase gene was confirmed by PCR after 20 passages in culture. A number of 25 animals, 10-week old *nu/nu* mice, obtained from Jackson Laboratories were injected directly into the kidney with luciferase-transduced hAFSC (1×10^6^ cells/mouse diluted in 50 µl ofPBS) after glycerol damage. *In vivo* optical imaging was performed with a prototype IVIS 3-dimensional bioluminescence/fluorescence optical imaging system [IVIS 100, Caliper Life Sciences, Hopkinton] at different time points.

Prior to imaging, each mouse was given an intraperitoneal injection of luciferin [Promega] at a dose of 125 mg/kg, as previously described [29]. As control, to exclude background signal, 5 mice were injected only with luciferin and no cells. General anesthesia was then induced with 5% isoflurane and the mouse was placed in the light-tight heated chamber; anesthesia was continued during the procedure with 1% isoflurane introduced via a nose cone. The imaging system consists of a cooled, back-thinned charge-coupled device (CCD) camera to capture both a visible light photograph of the animal taken with light-emitting diodes and a luminescent image. A rotating mirror and translatable animal stage allowed for images to be acquired over 360°.

### Immunostaining

Frozen and paraffin slides were stained for immunofluorescence. Paraffin slides were deparaffinized as previously described while the frozen slides were fixed for 5 minutes in 80% methanol. The slides were placed in 1% Triton x-100 [VWR] in PBS for 5 minutes (if the antigen was nuclear) and briefly washed in PBS. The slides were then placed in working solution of Vector Antigen Retrieval as described in the data sheet. [Vector Laboratories] for three cycles at high power for 4 minutes each afterwards cooled down at room temperature. After Avidin/Biotin blocking using the Blocking Avidin/Biotin Vector kit [Vector Laboratories] for 30 minutes at room temperature, a second block was carried out for 30 minutes using 5% of Bovine Serum Albumin [BSA, Sigma-Aldrich] diluted in PBS at room temperature. The slides were then incubated in primary antibody at different concentrations in solution a of 5% of BSA in PBS [Dolicholus Biflorus (conc. 1∶50) and Peanut Agglutinin (conc. 1∶50) form Vector Laboratories, Luciferase from Promega (conc. 1∶100) and Glial Derived Neurotrophic Factor (conc. 1∶50) and Aquaporin 2 (conc. 1∶50), Santa Cruz] solution for one hour at room temperature (Dolicholus Biflorus and Peanut Agglutinin) or overnight at 4°C (Luciferase and GDNF). Afterwards, the slides were washed in PBS for 5 minutes for 3 times. Secondary antibodies [Vector Laboratories] were diluted 1∶200 in 5% BSA in PBS – slides were incubated in this solution for 1.5 hours at room temperature, followed by washes in PBS for 5 minutes for 3 times. The appropriate fluorescent marker [Texas Red or Fluorescein Avidin DCS from Vector Laboratories] was then applied in a concentration of 1∶500 in PBS buffer for 5–10 minutes, followed by a final wash in PBS for 5 minutes for 3 times. Sections were counterstained with 4′,6-diamidino-2-phenylindole (DAPI) (Vector Laboratories).

A Leica DM RA fluorescent microscope was used in conjunction with Open Lab 3.1.5 software to image the staining.

### Apoptosis and Proliferation Measurements

The number apoptotic cells were determined using the TUNEL Apoptosis Detection Kit for paraffin-embedded tissue sections (Biotin-labeled POD; GenScript c# L00297) at 24 hours, 48 hours, 72 hours, 1 week and 2 weeks after injections in every experimental group (**1.** 10 control animals, no damage and no treatment; **2.** 10 animals that underwent only ATN; **3.** 10 animals that underwent ATN and intrarenal injection of hAFSC; **4.** 10 animals that underwent ATN and intrarenal injection of saline vehicle solution (PBS). **5.** 10 animals that underwent intrarenal injection of hAFSC but not ATN) as suggested by the kit data sheet. All the reagents were furnished by the kit. Briefly, after deparaffination and rehydration, the slides were incubated with Proteinase K solution for 30 minutes at 37°C, washed in PBS for 2 minutes, and after blocking incubated with a TUNEL Reaction Mixture for 1 hour at 37°C, followed by the Streptavidin –HRP solution for 30 minutes at 37°C and finally revealed with the DAB Substrate. If the staining was performed in frozen section the fluorescent TUNEL [In Situ Cell Death Detection kit, fluorescein; Roche, Applied-Science, c# 11684795910] was used. Briefly, the cells were incubated at 37°C for one hour with the TUNEL reagent and then washed in PBS. Slides were mounted with Vector DAPI mounting medium [Vector Laboratories].

In all the experimental groups, the apoptotic nuclei were counted as a fraction of the total number of nuclei present in the section using consecutive, non-overlapping fields of TUNEL-stained specimens. The percentage of apoptotic cells was estimated without knowledge of the experimental group.

The number of proliferative cells was determined using the PCNA Staining Kit (Invitrogen, c# 93–1143) at 24 hours, 48 hours, 72 hours, 1 week and 2 weeks after injections in every experimental group above described as suggested by the kit data sheet. Briefly, after deparaffination and rehydration and blocking, the slides were incubated with a biotinylated mouse anti-PCNA primary antibody for 1 hour at room temperature followed with incubation with Streptevidin-HRP and revealed with DAB substrate. In all the experimental groups the proliferative nuclei were counted as a fraction of the total number of nuclei present in the section using consecutive, non-overlapping fields of PCNA-stained specimens. The percentage of proliferative cells was estimated without knowledge of the experimental group.

### Blood Collection, Creatinine and BUN Measurements

The facial vein was lanced with a 5 mm animal lancet and blood collected using standard protocols approved by the Animal Core Facility at Childrens Hospital of Los Angeles and Saban Research Institute. Animals were divided into different groups per each time point as follow (24 hours, 48 hours, 72 hours, 1 week and 2 weeks): **1.** 10 animals for measuring baseline creatinine and BUN levels; **2.** 10 animals that underwent only ATN; **3.** 10 animals that underwent ATN and intrarenal injection of hAFSC; **4.** 10 animals that underwent ATN and intrarenal injection of saline vehicle solution (PBS). **5.** 10 animals that underwent intrarenal injection of hAFSC but not ATN.

The blood samples (30 µL) were collected into plasma separation tubes with lithium heparin. They were centrifuged at 13,000-RPM for 3 minutes and the plasma (upper layer) was removed and stored at −80°C until analysis. A maximum of 15% of circulating blood was sampled in a given 14-day period (total blood volume ∼0.6% of total body weight). Post-damage measurements were obtained every 24 hours. The blood samples were used to monitor renal function, by analyzing creatinine and BUN levels. Expression levels were determined by a quantitative colorimetric assay [BioAssay Systems Cat # DUCT-500 for creatinine Cat # DIUR-500 for BUN], using an improved Jaffe method for creatinine, and an improved Jung method for BUN without any pretreatment of the samples.

### Morphological Studies

Paraffin embedded kidney sections were prepared at 4 µm thickness by a routine procedure and stained with PAS reagents as described above. The kidney sections were divided as follow: **1.** Sections obtained from mice that underwent ATN with no injection of hAFSC sacrificed at 24 hours, 48 hours, 72 hours, 1 week and 2 weeks; **2.** Section obtained from mice that underwent ATN and injection of hAFSC sacrificed at 24 hours, 48 hours, 72 hours, 1 week and 2 weeks; **3.** Sections obtained from mice that underwent ATN with and injection of PBS sacrificed at 24 hours, 48 hours, 72 hours, 1 week and 2 weeks; **4.** Sections obtained from mice that underwent only intrarenal injection of hAFSC and sacrificed at 24 hours, 48 hours, 72 hours, 1 week and 2 weeks. Tubular injury was evaluated based on different parameters using PAS staining: including disruption of tubular membranes and brush borders, but mainly relaying on cast formation since it is the most evident sign of damage in stained sections.

In the experimental groups, the tubular injury was counted as a fraction of the total number of tubules present in the section using consecutive, non-overlapping fields of PAS-stained specimens. The percentage of damaged tubules was estimated without knowledge of the experimental group.

### Cytokine Analysis

To examine if the injection of hAFSC would interfere with the modulation of inflammation after acute kidney injury human and mouse cytokines levels were measured in digested mouse kidneys at 24 hours, 48 hours, 72 hours, 1 week and 2 weeks in every experimental groups (**1.** control animals, no damage and no treatment; **2.** animals that underwent only ATN; **3.** animals that underwent ATN and intrarenal injection of hAFSC; **4.** animals that underwent ATN and intrarenal injection of saline vehicle solution (PBS). **5.** animals that underwent intrarenal injection of hAFSC but not ATN) using a multiple cytokine array technique Proteome Profiler Array Kit, for human c# ARY005 and for mouse c# ARY006, as suggested from the protocol [R&D Systems].

Kidneys are briefly washed in PBS, cut in pieces and added to 1 ml Phosphatase Inhibitor solution [ActiveMotif] and then mechanically homogenized on ice for 1 minute, then 10 µl of Triton X-100 [Sigma-Aldrich] is added before vortexing for 1 minute. The lysate is frozen at -80°C and thawed at room temperature. The sample is centrifuged at 10.000 rpm for 20 minutes at 4°C. The supernatant is then transferred to a clean tube and the protein concentration measured by UV-vis spectroscopy. An equal amount of protein (300 µg) for the samples derived form the different experimental groups (for hAFSC cultured *in vitro* to test the basal level of cytokines expression before *in vivo* injection, the amount of protein was extracted from 1×10^6^ cells, the same amount used for injection) was incubated for one hour at room temperature with a cytokine cocktail and each sample was then added to the membrane and incubated overnight at 4°C. The following day after several washing steps in Washing Buffer the membranes were incubated with a streptavidin-HRP secondary as suggested by the manufacturer (concentration 1∶2000) for 30 minutes at room temperature. After washing in Washing buffer, it was added the Super Signal West Pico Chemiluminescent Substrate was added [ThermoScientific] to detect the signal. After 1 minute incubation the membranes were exposed for 20 seconds to x-ray film. The signal was measured by counting the pixel density and the data were analyzed using the Array Vision Program [R&D Systems].

### Statistical Analysis

All graphical data are displayed as the mean + SEM for n animals in the number of independent experiments. For cytokines one-way analysis of variance (ANOVA) with Bonferroni post-test correction was used, and for all other data Mann-Whitney U test was applied to compare two ore more (ANOVA) independent sets of data. A P value less than 0.05 was considered statistically significant.

## Supporting Information

Table S1 In the table are reported the P values for the cytokine analysis at 1,2, 3, 7 and 14 days. Column 1: Cytokines are grouped by target and/or effects. Column 2: Mice with ATN and injection of hAFSC versus normal nu/nu mouse (cytokine basal levels before any treatment). Column 3: Mice with ATN and injection of PBS versus normal nu/nu mouse (cytokines basal levels before any treatment). Column 4: Mice with ATN only versus normal nu/nu mouse (cytokines basal levels before any treatment). Column 5: Mice with no ATN and injection of hAFSC versus normal nu/nu mouse (cytokines basal level before any treatment). P values are expressed as follows: * P<0.05; ** P < 0.01; *** P< 0.001 and they represent the deviation from the control (normal nu/nu mouse); ⇑: increase of cytokine levels in the experimental groups compared to the control; ⇓: decrease of cytokine levels in the experimental groups compared to the control. Blank cells in the table indicate no statistically significant change in cytokine expression.
(0.08 MB DOC)

Table S2 In the table are reported the P values for the mouse cytokine analysis at 1, 2, 3, 7 and 14 days. Column 1: Cytokines are grouped by target and/or effects. Column 2: Mice with ATN and injection of hAFSC versus mice with ATN and injection of PBS (⇑: increase of cytokine levels in mice with ATN and injection of hAFSC versus mice with ATN and injection of PBS; ⇓: decrease of cytokine levels in mice with ATN and injection of hAFSC versus mice with ATN and injection of PBS). Column 3: Mice with ATN and injection of hAFSC versus mice with ATN only (⇑: increase of cytokine levels in mice with ATN and injection of hAFSC versus mice with ATN only; ⇓: decrease of cytokine levels in mice with ATN and injection of hAFSC versus mice with ATN only). Column 4: Mice with ATN and injection of PBS versus mice with ATN only (⇑: increase of cytokine levels in mice with ATN and injection of PBS versus mice with ATN only; ⇓: decrease of cytokine levels in mice with ATN and injection of PBS versus mice with ATN only). P values are expressed as follows: * P < 0.05, ** P < 0.01, *** P< 0.001. Blank cells in the table indicate no statistically significant change in cytokine expression.
(0.08 MB DOC)

Table S3 In the table are reported the P values for the human cytokine analysis at 1, 2, 3, 7 and 14 days. Column 1: Cytokines are grouped by target and/or effects. Column 2: Mice with ATN and injection of hAFSC versus hAFSC in vitro (hAFSC cytokines basal level) (⇑: increase of human cytokine levels in mice with ATN and injection of hAFSC versus hAFSC in vitro; ⇓: decrease of cytokine levels in mice with ATN and injection of hAFSC versus hAFSC in vitro). Column 3: Mice without ATN and injection of hAFSC versus hAFSC in vitro (hAFSC cytokines basal level) (⇑: increase of human cytokine levels in mice without ATN and injection of hAFSC versus hAFSC in vitro; ⇓: decrease of cytokine levels in mice without ATN and injection of hAFSC versus hAFSC in vitro). P values are expressed as follows: * P < 0.05, ** P < 0.01, *** P< 0.001. Blank cells in the table indicate no statistically significant change in cytokine expression.
(0.06 MB DOC)
