# Supplementary material for: Protective Effect of Human Amniotic Fluid Stem Cells in an Immunodeficient Mouse Model of Acute Tubular Necrosis
Source: PLoS One. 2010 Feb 24;5(2):e9357. doi: 10.1371/journal.pone.0009357 (PMC2827539; doi:10.1371/journal.pone.0009357)
Supplement: Table S1 — In the table are reported the P values for the cytokine analysis at 1,2, 3, 7 and 14 days. Column 1: Cytokines are grouped by target and/or effects. Column 2: Mice with ATN and injection of hAFSC versus normal nu/nu mouse (cytokine basal levels before any treatment). Column 3: Mice with ATN and injection of PBS versus normal nu/nu mouse (cytokines basal levels before any treatment). Column 4: Mice with ATN only versus normal nu/nu mouse (cytokines basal levels before any treatment). Column 5: Mice with no ATN and injection of hAFSC versus normal nu/nu mouse (cytokines basal level before any treatment). P values are expressed as follows: * P<0.05; ** P < 0.01; *** P< 0.001 and they represent the deviation from the control (normal nu/nu mouse); ⇑: increase of cytokine levels in the experimental groups compared to the control; ⇓: decrease of cytokine levels in the experimental groups compared to the control. Blank cells in the table indicate no statistically significant change in cytokine expression. (0.08 MB DOC) [file pone.0009357.s001.doc]

**Table S1: Statistical significance (P values) of expression of mouse cytokines at 1, 2, 3, 7 and 14 days in all experimental groups compared to the mouse physiological cytokine level**

| MOUSE  CYTOKINES | Mice with ATN and injection of hAFSC  Normal *nu/nu* Mouse | Mice with ATN and injection of PBS  Normal *nu/nu* Mouse | Mice with ATN only  Normal *nu/nu* Mouse | | Mice injected with hAFSC and no ATN  Normal *nu/nu* Mouse |
| --- | --- | --- | --- | --- | --- |
| **Interleukins** | | | | | |
| IL-1α | **, 14 days post inj. ↓ |  | |  | *, 3 days post inj. ↓  *, 7 days post inj. ↑ |
| IL-1β | *, 14 days post inj. ↓ |  | |  |  |
| IL-2 |  | *, 14 days post inj. ↑ | | *, 3 days post inj. ↑  **, 7 days post inj.↑ |  |
| IL-13 | *, 3 days post inj. ↓  *, 7 days post inj. ↓  *, 14 days post inj. ↓ | *, 2 days post inj. ↓ | |  | *, 7 days post inj. ↑ |
| IL-12p70 |  | *, 14 days post inj. ↑ | | *, 2 days post inj. ↑ | **, 2 days post inj. ↑  *, 7 days post inj. ↑  *, 14 days post inj. ↑ |
| IL-16 | **, 1 days post inj. ↓  **, 14 days post inj. ↓ | **, 7 days post inj. ↑  *, 14 days post inj. ↑ | |  | *, 1 days post inj. ↓  *, 7 days post inj. ↑ |
| IL-23 |  | *, 7 days post inj. ↑  **, 14 days post inj. ↑ | | *, 1 days post inj. ↑  **, 7 days post inj.↑ | *, 2 days post inj. ↑  *, 7 days post inj. ↑  *, 14 days post inj. ↑ |
| IL-27 |  | **, 14 days post inj. ↑ | | ***, 7 days post inj. ↑ | *, 2 days post inj. ↑  *, 3 days post inj. ↑ |
| IL-1ra | ***, 2 days post inj. ↑  ***, 7 days post inj. ↑ | *, 2 days post inj. ↑  ***, 7 days post inj. ↑  ***, 14 days post inj. ↑ | | *, 1 days post inj. ↑  *, 3 days post inj. ↑  *, 7 days post inj. ↑ | *, 7 days post inj. ↑ |
| IL-6 | **, 14 days post inj. ↑ |  | |  | *, 7 days post inj. ↑ |
| IL-10 | *, 14 days post inj. ↑ |  | |  | *, 7 days post inj. ↑ |
| **Activators of Lymphocytes B** | | | | | |
| BLC |  |  |  | | *, 14 days post inj. ↑ |
| SDF-1 |  | *, 3 days post inj. ↑  **, 14 days post inj. ↑ | *, 1 days post inj. ↑  ***, 3 days post inj. ↑  *, 7 days post inj. ↑ | |  |
| **Activators of Natural Killers** | | | | | |
| IP-10 | *, 3 days post inj. ↓  *, 14 days post inj. ↓ |  |  | | *, 3 days post inj. ↓ |
| IL-27 |  | **, 14 days post inj. ↑ | ***, 7 days post inj. ↑ | | *, 2 days post inj. ↑  *, 3 days post inj. ↑ |
| MIG | *, 14 days post inj. ↓ |  |  | |  |
| JE | **, 2 days post inj. ↑ | **, 7 days post inj. ↑ | **, 3 days post inj. ↑ | |  |
| **Chemotactic Attractors of Granulocytes and Macrophages** | | | | | |
| C5a |  |  |  | |  |
| G-CSF | *, 3 days post inj. ↓  *, 14 days post inj. ↓ |  |  | |  |
| KC | *, 2 days post inj. ↑ | **, 2 days post inj. ↑  *, 14 days post inj. ↑ | *, 2 days post inj. ↑  **, 3 days post inj. ↑  *, 14 days post inj. ↑ | |  |
| M-CSF |  |  | *, 14 days post inj. ↑ | |  |
| JE |  | **, 2 days post inj. ↑ | **, 7 days post inj. ↑ | | **, 3 days post inj. ↑ |
| MCP-5 | *, 14 days post inj. ↓ |  |  | | *, 3 days post inj. ↓ |
| I-309 |  |  | **, 7 days post inj. ↑ | |  |
| MIP-2 |  |  | *, 3 days post inj. ↑ | |  |
| RANTES | **, 2 days post inj. ↑  **, 14 days post inj. ↓ | *, 7 days post inj. ↑  **, 14 days post inj. ↑ | **, 3 days post inj. ↑  *, 7 days post inj. ↑ | | ***, 2 days post inj. ↑  *, 14 days post inj. ↑ |
| SDF-1 |  | *, 3 days post inj. ↑  **, 14 days post inj. ↑ | *, 1 days post inj. ↑  ***, 3 days post inj. ↑  *, 7 days post inj. ↑ | |  |
| **Multiple Biological Effectors** | | | | | |
| TNF-α |  |  |  | |  |
| MIP-1α | **, 14 days post inj. ↓ | *, 1 day post inj. ↑ | **, 7 days post inj. ↑ | | *, 7 days post inj. ↑  *, 14 days post inj. ↑ |
| MIP-1β | *, 7 days post inj. ↓  **, 14 days post inj. ↓ | *, 2 days post inj. ↓ |  | | *, 3 days post inj. ↓ |
| TIMP-1 | **, 14 days post inj. ↓ | **, 2 days post inj. ↑  **, 3 days post inj. ↑  *, 7 days post inj. ↑  *, 14 days post inj. ↑ | **, 2 days post inj. ↑  *, 14 days post inj. ↑ | |  |
| sICAM-1 | **, 14 days post inj. ↓ | *, 7 days post inj. ↑  **, 14 days post inj. ↑ | **, 2 days post inj. ↑  **, 3 days post inj. ↑  **, 7 days post inj. ↑  *, 14 days post inj. ↑ | | *, 7 days post inj. ↑ |
