# Supplementary material for: Protective Effect of Human Amniotic Fluid Stem Cells in an Immunodeficient Mouse Model of Acute Tubular Necrosis
Source: PLoS One. 2010 Feb 24;5(2):e9357. doi: 10.1371/journal.pone.0009357 (PMC2827539; doi:10.1371/journal.pone.0009357)
Supplement: Table S2 — In the table are reported the P values for the mouse cytokine analysis at 1, 2, 3, 7 and 14 days. Column 1: Cytokines are grouped by target and/or effects. Column 2: Mice with ATN and injection of hAFSC versus mice with ATN and injection of PBS (⇑: increase of cytokine levels in mice with ATN and injection of hAFSC versus mice with ATN and injection of PBS; ⇓: decrease of cytokine levels in mice with ATN and injection of hAFSC versus mice with ATN and injection of PBS). Column 3: Mice with ATN and injection of hAFSC versus mice with ATN only (⇑: increase of cytokine levels in mice with ATN and injection of hAFSC versus mice with ATN only; ⇓: decrease of cytokine levels in mice with ATN and injection of hAFSC versus mice with ATN only). Column 4: Mice with ATN and injection of PBS versus mice with ATN only (⇑: increase of cytokine levels in mice with ATN and injection of PBS versus mice with ATN only; ⇓: decrease of cytokine levels in mice with ATN and injection of PBS versus mice with ATN only). P values are expressed as follows: * P < 0.05, ** P < 0.01, *** P< 0.001. Blank cells in the table indicate no statistically significant change in cytokine expression. (0.08 MB DOC) [file pone.0009357.s002.doc]

**Table S2: Statistical significance (P values) of mouse cytokines data at 1, 2, 3, 7 and 14 days between mice that underwent ATN plus injection of hAFSC, mice that underwent ATN plus injection of PBS and mice that went only ATN injection.**

| MOUSE  CYTOKINES | Mice with ATN and injection of hAFSC  Mice with ATN and injection of PBS | Mice with ATN and injection of hAFSC  Mice with ATN only | Mice with ATN and injection of PBS  Mice with ATN only |
| --- | --- | --- | --- |
| **Interleukins** | | | |
| IL-1α | ***, 14 days post inj. ↓ | **, 14 days post inj. ↓ |  |
| IL-1β | ***, 14 days post inj. ↓ | *, 14 days post inj. ↓ |  |
| IL-2 | ***, 14 days post inj. ↓ | *, 14 days post inj. ↓ | **, 14 days post inj. ↑ |
| IL-13 | **, 14 days post inj. ↓ | *, 7 days post inj. ↑  *, 14 days post inj. ↓ |  |
| IL-12p70 | **, 14 days post inj. ↓ |  |  |
| IL-16 | **, 14 days post inj. ↓ | **, 1 days post inj. ↓  *, 14 days post inj. ↓ |  |
| IL-23 | **, 14 days post inj. ↓ |  | *, 14 days post inj. ↑ |
| IL-27 | **, 14 days post inj. ↓ | *, 14 days post inj. ↓ |  |
| IL-1ra | ***, 14 days post inj. ↓ |  |  |
| IL-6 | *, 14 days post inj. ↓ | **, 14 days post inj. ↓ |  |
| IL-10 | *, 14 days post inj. ↓ | *, 7 days post inj. ↓  **, 14 days post inj. ↓ | *, 14 days post inj. ↓ |
| **Activators of Lymphocytes B** | | | |
| BLC | **, 14 days post inj. ↓ | **, 14 days post inj. ↓ |  |
| SDF-1 | **, 14 days post inj. ↓ | *, 7 days post inj. ↓ | *, 3 days post inj. ↓  **, 14 days post inj. ↑ |
| **Activators of Natural Killers** | | | |
| IP-10 |  | *, 14 days post inj. ↓ |  |
| IL-27 | **, 14 days post inj. ↓ | *, 14 days post inj. ↓ |  |
| MIG | **, 14 days post inj. ↓ | *, 7 days post inj. ↓  *, 14 days post inj. ↓ |  |
| JE | *, 7 days post inj. ↓  *, 14 days post inj. ↓ |  | *, 7 days post inj. ↑ |
| **Chemotactic Attractors of Granulocytes and Macrophages** | | | |
| C5a | *, 14 days post inj. ↓ | *, 14 days post inj. ↓ |  |
| G-CSF | *, 14 days post inj. ↓ | ***, 14 days post inj. ↓ |  |
| Eotaxin | **, 14 days post inj. ↓ | *, 14 days post inj. ↓ |  |
| KC | **, 14 days post inj. ↓ | **, 14 days post inj. ↓ |  |
| M-CSF | ***, 14 days post inj. ↓ | ***, 14 days post inj. ↓ |  |
| JE | *, 7 days post inj. ↓  *, 14 days post inj. ↓ |  | *, 7 days post inj. ↑ |
| MCP-5 | *, 14 days post inj. ↓ | *, 7 days post inj. ↓  **, 14 days post inj. ↓ |  |
| I-309 | **, 14 days post inj. ↓ | ***, 7 days post inj. ↓  **, 14 days post inj. ↓ | *, 7 days post inj. ↓ |
| MIP-2 | *, 7 days post inj. ↓  **, 14 days post inj. ↓ | *, 7 days post inj. ↓ |  |
| RANTES | ***, 14 days post inj. ↓ | *, 14 days post inj. ↓ | **, 14 days post inj. ↑ |
| SDF-1 | **, 14 days post inj. ↓ | *, 7 days post inj. ↓ | *, 3 days post inj. ↓  **, 14 days post inj. ↑ |
| **Multiple Biological Effectors** | | | |
| TNF-α | *, 14 days post inj. ↓ | **, 14 days post inj. ↓ |  |
| IFN-γ | **, 14 days post inj. ↓ | **, 14 days post inj. ↓ | *, 2 days post inj. ↓ |
| MIP-1α | **, 14 days post inj. ↓ | *, 14 days post inj. ↓ |  |
| MIP-1β | **, 14 days post inj. ↓ | **, 7 days post inj. ↓  ***, 14 days post inj. ↓ |  |
| TIMP-1 | *, 2 days post inj. ↓  ***, 14 days post inj. ↓ | *, 2 days post inj. ↓  **, 14 days post inj. ↓ |  |
| sICAM-1 | *, 7 days post inj. ↓  **, 14 days post inj. ↓ | **, 7 days post inj. ↓  **, 14 days post inj. ↓ |  |
