# Supplementary material for: Protective Effect of Human Amniotic Fluid Stem Cells in an Immunodeficient Mouse Model of Acute Tubular Necrosis
Source: PLoS One. 2010 Feb 24;5(2):e9357. doi: 10.1371/journal.pone.0009357 (PMC2827539; doi:10.1371/journal.pone.0009357)
Supplement: Table S3 — In the table are reported the P values for the human cytokine analysis at 1, 2, 3, 7 and 14 days. Column 1: Cytokines are grouped by target and/or effects. Column 2: Mice with ATN and injection of hAFSC versus hAFSC in vitro (hAFSC cytokines basal level) (⇑: increase of human cytokine levels in mice with ATN and injection of hAFSC versus hAFSC in vitro; ⇓: decrease of cytokine levels in mice with ATN and injection of hAFSC versus hAFSC in vitro). Column 3: Mice without ATN and injection of hAFSC versus hAFSC in vitro (hAFSC cytokines basal level) (⇑: increase of human cytokine levels in mice without ATN and injection of hAFSC versus hAFSC in vitro; ⇓: decrease of cytokine levels in mice without ATN and injection of hAFSC versus hAFSC in vitro). P values are expressed as follows: * P < 0.05, ** P < 0.01, *** P< 0.001. Blank cells in the table indicate no statistically significant change in cytokine expression. (0.06 MB DOC) [file pone.0009357.s003.doc]

**Table S3: Statistical significance (P values) of human cytokines data at 1, 2, 3, 7 and 14 days in all mice injected with or without ATN compared to the hAFSC basal cytokine levels *in vitro***

| HUMAN  CYTOKINES | Mice with ATN and injection of hAFSC  Normal cells | Mice injected with hAFSC and no ATN  Normal cells |
| --- | --- | --- |
| **Interleukins** | | |
| IL-1α | *, 1 days post inj. ↑  *, 3 days post inj. ↑  *, 7 days post inj. ↑ | *, 3 days post inj. ↑  **, 7 days post inj. ↑ |
| IL-1β | **, 1 day post inj. ↑  **, 7 days post inj. ↑ | *, 1 day post inj. ↑  **, 7 days post inj. ↑ |
| IL-2A | **, 1 day post inj. ↑  *, 3 days post inj. ↑  **, 7 days post inj. ↑ | *, 1 days post inj. ↑  **, 7 days post inj. ↑ |
| IL-13 | *, 1 day post inj. ↑  *, 3 days post inj. ↑  **, 7 days post inj. ↑ | *, 3 days post inj.↑  *, 7 days post inj. ↑ |
| IL-12p70 | **, 1 day post inj. ↑  ***, 7 days post inj. ↑ | *, 1 day post inj. ↑  *, 3 days post inj. ↑  **, 7 days post inj. ↑ |
| IL-16 | **, 7 days post inj. ↑  *, 14 days post inj. ↑ | *, 3 days post inj. ↑  *, 7 days post inj. ↑ |
| IL-23 | ***, 7 days post inj. ↑ | *, 3 days post inj. ↑  **, 7 days post inj. ↑ |
| IL-27 | *, 7 days post inj. ↑ | *, 3 days post inj. ↑  *, 7 days post inj. ↑ |
| IL-1ra | *, 1 day post inj. ↑  ***, 7 days post inj. ↑ | *, 3 days post inj. ↑  **, 7 days post inj. ↑ |
| IL-6 | *, 1 day post inj. ↓  **, 2 days post inj. ↓  *, 3 days post inj. ↓  *, 7 days post inj. ↓ | **, 1 day post inj. ↓  ***, 3 days post inj. ↓  **, 7 days post inj. ↓ |
| IL-10 | ***, 1 day post inj. ↑  ***, 7 days post inj. ↑ | *, 1 day post inj. ↑  *, 3 days post inj. ↑  *, 7 days post inj. ↑ |
| **Activators of Lymphocytes B** | | |
| BLC |  |  |
| SDF-1 | *, 1 day post inj. ↑  ***, 7 days post inj. ↑ | *, 1 day post inj. ↑  **, 7 days post inj. ↑ |
| **Activators of Natural Killers** | | |
| IP-10 | **, 1 day post inj. ↑  ***, 7 days post inj. ↑ | *, 1 day post inj. ↑  **, 3 days post inj. ↑  *, 7 days post inj. ↑ |
| IL-27 | *, 7 days post inj. ↑ | *, 3 days post inj. ↑  *, 7 days post inj. ↑ |
| **Chemotactic Attractors of Granulocytes and Macrophages** | | |
| G-CSF | **, 1 day post inj. ↑  **, 7 days post inj. ↑ | *, 7 days post inj. ↑ |
| I-309 | **, 7 days post inj. ↑ | *, 3 days post inj. ↑  **, 7 days post inj. ↑ |
| RANTES | *, 1 day post inj. ↑  *, 2 days post inj. ↑  **, 7 days post inj. ↑ | *, 1 day post inj. ↑  **, 3 days post inj. ↑  **, 7 days post inj. ↑ |
| GRO-α |  |  |
| SDF-1 | *, 1 day post inj. ↑  ***, 7 days post inj. ↑ | *, 1 day post inj. ↑  **, 7 days post inj. ↑ |
| **Multiple Biological Effectors** | | |
| TNF-α | *, 1 day post inj. ↑ | *, 1 day post inj. ↑  *, 7 days post inj. ↑ |
| IFN-α | **, 7 days post inj. ↑ | *, 3 days post inj. ↑  *, 7 days post inj. ↑ |
| MIP-1α | *, 1 day post inj. ↑  *, 3 days post inj. ↑  ***, 7 days post inj. ↑ | **, 1 day post inj. ↑  *, 3 days post inj. ↑  *, 7 days post inj. ↑ |
| MIP-1β | *, 1 day post inj. ↑ |  |
| sICAM-1 | *, 1 day post inj. ↑  ***, 7 days post inj. ↑ | *, 3 days post inj. ↑  **, 7 days post inj. ↑ |
| GMCSF |  | **, 7 days post inj. ↑ |
| MCP-1 |  |  |
| MIF |  | *, 1 day post inj. ↓  *, 3 days post inj. ↓  **, 7 days post inj. ↓ |
